# Physical Activity of Workers in a Hospital

**DOI:** 10.3390/ijerph16040532

**Published:** 2019-02-13

**Authors:** So Yeon Jun, Jaewon Kim, Hyehoon Choi, Joon Sung Kim, Seong Hoon Lim, Bomi Sul, Bo Young Hong

**Affiliations:** Department of Rehabilitation Medicine, St. Vincent’s Hospital, College of Medicine, The Catholic University of Korea, Seoul 16247, Korea; iamsj17@naver.com (S.Y.J.); jw2356@naver.com (J.K.); choihyehoon1021@gmail.com (H.C.); svpmr@catholic.ac.kr (J.S.K.); seonghoon@catholic.ac.kr (S.H.L.); snowspringee@naver.com (B.S.)

**Keywords:** occupational health, physical activity, healthcare worker

## Abstract

*Purpose*: This study aims to evaluate the physical activity of healthcare personnel and the affecting factors of physical activity (PA) in a hospital using an accelerometer device (Actigraph wGT3X-BT). *Method*: A total of 63 subjects (22 physicians, 19 nurses, and 23 supporting staff) participated and wore an accelerometer for seven days. Among the outputs, the mean counts for a minute, time spent for light, moderate, and vigorous intensity PA, and step count were extracted. As a secondary study, 16 subjects continued for one more week after feedback on their PA of the previous week and counseling to encourage PA. *Result*: Most of (62/63) the participants fulfilled the recommended amount of PA, which is more than 300 min of moderate to vigorous physical activity (MVPA). Physicians showed significantly less PA than nurses or supporting staffs: Mean counts per minute (210.4 vs. 476.0 and 441.8 respectively), time in MVPA per week (904.7 min vs. 1471.3 min and 1451.0 min), and step counts per week (69,029 vs. 87,119 and 84,700) (*p* < 0.001). Nurses and supporting staff were not statistically different. There was no significant difference in the PA of workers in the hospital regarding gender and marital status. However, the average calorie expenditure of the child raising group was significantly higher. There was no statistically significant difference in PA before and after counseling. No participants reported a vigorous degree of exercise intensity over the study period. *Conclusion*: Most of the healthcare personnel met the recommended PA, however, only 57% (36/63) recalled having engaged in MVPA during the study period. The group of physicians showed less PA compared to nurses or supporting staff. Single check-up and counseling were not found to increase PA.

## 1. Background

Physical activity (PA) is defined as any bodily movement produced by skeletal muscles that requires energy expenditure [1]. PA, especially moderate to vigorous intensity PA (MVPA), is highly recommended for preventing cardiovascular diseases, type-2 diabetes, some kinds of cancers, and improving the quality of life [2,3]. The amount of PA has a dose–response relationship with all causes of mortality and cardiovascular diseases [4]. Additionally, PA is shown to counter depression, anxiety disorders, and other mood dysfunctions [5,6,7]. Therefore, interest in the benefits of PA on specific diseases, health, and well-being has increased, and much research is ongoing [8].

The World Health Organization (WHO) provided recommendations for PA to benefit health in 2010. These recommendations stated that for additional health benefits, adults should increase their moderate intensity aerobic PA to 300 min per week, or engage in 150 min of vigorous intensity aerobic PA per week, or an equivalent combination of moderate and vigorous intensity activity [1].

Nevertheless, the levels of physical inactivity are rising in many countries and have major implications for the general health of the population worldwide [9,10]. About 31% (range from 17% in South-East Asia to 43% in the Americas and Eastern Mediterranean) of overall adults, worldwide, showed physical inactivity [1,11]. The proportion of Korean adults who are regular exercisers is steadily decreasing, and less than 30% of adults are estimated to be doing the recommended amount of PA [12].

Healthcare personnel endure heavy workloads in emotionally stressful conditions, and they are prone to musculoskeletal problems, psychiatric disorders, such as depression or burnout, and a lower health-related quality of life [13,14,15]. We assumed that healthcare personnel were at risk of physical inactivity due to the lack of leisure time PA. Owoeye el al. measured the step count using a pedometer among nurses, pharmacists, and physiotherapists and the mean 7396.94 ± 2714.63 steps/day was revealed, which was less than the generally accepted recommendation, 10,000 steps/day [16]. However, unlike accelerometer, pedometer does not accurately reflect energy expenditure or intensity of PA [17]. Furthermore, in most recommendations, it is marked as a time of MVPA, which is different from the number of steps. In addition, the previous study did not involve physicians—one of the essential occupations of healthcare workers. The primary goal of this study was to evaluate objectively the amount of PA of healthcare personnel using an accelerometer to find out which factors influence the PA level. In addition, we planned to find out whether there is any improvement in the level of PA when people receive counseling to encourage PA and informative feedback on their PA. To our knowledge, this is the first study to present the PA of healthcare personnel including physicians using an accelerometer, which is more objective than a questionnaire.

## 2. Methods

### 2.1. Study Population

This study was performed at one university hospital from 1 May to 31 August 2017. An institutional review board reviewed the study protocol (VC170ESI0101), and informed consent was obtained from each subject. The inclusion criteria of our study were those (1) who work in the hospital as a physician, nurse, or supporting staff, such as radiographers or patient-transfer workers, etc., (2) aged from 19 to 60 years, (3) who agreed to participate with informed consent. Persons with fractures or other serious medical conditions or social circumstances that limit PA, or those who have two or more days off-duty during one week of study were excluded.

### 2.2. Measures

We used Actigraph wCT3x-BT (ActiGraph, Pensacola, FL, USA) to monitor PA. Actigraph wGT3X-BT is a small accelerometer; it weighs about 19 g and measures the amount of PA objectively by recording the acceleration of the body movement. The wGT3X-BT has been widely used to capture and record continuous PA. It measures raw acceleration, the number of activity counts carried out, calorie expended, time spent according to intensity of activity; light, moderate, vigorous, and very vigorous intensity PA, average metabolic equivalent of tasks (METs) per hour, the number of steps taken, total device wear time, sleep latency, total hours of sleep, and sleep efficiency.

The participants were told to wear the accelerometer on the wrist of the non-dominant hand for the entire study period all day long including sleep time, except during water-based activities, such as bathing or swimming. It was given on the first day, and the subjects made an appointment to return the device and see the physician to discuss the analyzed results and to receive counseling about the PA on the seventh day. Demographic information was collected with participants’ questionnaires on their age, gender, height, weight, occupation, marital status, having kids or not, medical history, and smoking status. On the seventh day, each participant was asked about estimated total wear time per day, how many days, what exercise, and which intensity they did work out, to improve the accuracy of wear time and PA measurement. This was for additional validation of wear time and for comparing the accelerometer measurement with the subjectively perceived amount and intensity of physical activity. A simplified form of Korean-version of the International Physical Activity Questionnaire (IPAQ-K) for adults (15–69 years) was used for the questionnaire regarding exercise [18,19]. After that, the participants were told about their report of PA status and informed about the WHO recommendation on PA and the evidence-based effects of PA and exercise from a physician. After one-on-one counseling, they were encouraged to be more active and increase PA. After the completion of counseling, they were asked whether they wished to continue for an additional week to check for an increment in PA. If they agreed to continue, they were given the device for the additional week of recording. Participants were supposed to meet the researcher again after a week to discuss whether their PA had increased.

### 2.3. Data Analysis

After the study period, each collected data was extracted and analyzed through the ActiLife software version 6.13.3 (ActiGraph, Pensacola, FL, USA). We adopted the calculation of estimated energy expenditure using the method developed by Freedson et al., 1998 [20]. The activity count collected by accelerometer can be converted as MET (= 1.439008 + (0.000795 × counts/min)). Using the cut-point of MET (light < 3.0, 3.0 ≤ moderate < 6.0, 6.0 ≤ vigorous < 9.0, very vigorous ≥ 9.0), each activity count was interpreted as light (<1952 counts), moderate (1952–5724 counts), vigorous (5725–9498 counts), and very vigorous (>9498 counts) activity. A sixty seconds epoch was used. The length of bouts was a minimum of 10 consecutive minutes with allowed a drop time of 2 min. Non-wear was defined as a minimum length of 60 min with an allowed spike level to stop at 100 counts per minute and spike tolerance of 2 min. Wear time was validated using wear time validation tool in ActiLife software and each participants’ questionnaire after study. The information on PA, such as the sum of activity counts, time spent in sedentary, light, moderate, vigorous, and very vigorous intensity of PA, maximum average MET per hour, calorie expended per hour, total step count, total wear time, and sleep information were collected.

The participants were categorized into subgroups according to gender, marital status, body mass index (BMI, kg/m^2^), having children or not, and types of work (physicians, nurses, and supporting staff) to analyze the information on PA. Using the WHO Asia-Pacific region classification of BMI in adult Asians, BMI was classified as underweight (BMI < 18.5), normal (18.5 ≤ BMI < 23), and overweight (BMI ≥ 23) [21].

### 2.4. Statistical Analysis

The independent *t*-test and one-way ANOVA were used for the normal distribution and the Kruskal–Wallis test was used for the non-normal distribution (subgroups based on occupation or BMI). Regarding marital status, gender, and having kids, an independent *t*-test was used. Maximal average MET per hour score and total sleep time according to the occupation were analyzed with one-way ANOVA. Comparison of data after counseling with initial study, paired t-test was used if parameters are normally distributed and Wilcoxon signed rank test if parameters are not normally distributed. Paired *t*-test was used for mean counts per minute, maximum average MET per hour, light and moderate intensity activity per minute, and average steps count per minute. Wilcoxon signed rank test was used for total wear time and average calorie expended per hour. To identify the correlation between total sleep time and MVPA, Pearson correlation coefficient was used. SPSS version 21.0 (IBM, Armonk, NY, USA) was used and *p* < 0.05 was considered statistically significant.

## 3. Results

A total of 65 participants were recruited and enrolled for the study. We set the minimal device wear time at more than 10 hours per day except sleep time according to the frequently accepted minimal wear time [22,23,24]. Two participants were excluded because of insufficient device wear time. All the devices were returned after the study, and no equipment was proven to have any defect. The demographic characteristics of the participants are presented in Table 1. Among the 63 final participants, 22 were physicians, 18 were nurses, and 23 were supporting staff. The mean age was 35.83 ± 9.13 years old (range 24–57 years old). Thirty-one were men (32 women) and 32 participants were married. Thirty participants raised a child or children. No participant suffers from a serious medical condition. Among them, 5 had hypertension, 1 had type 2 diabetes mellitus, and 1 had hyperthyroidism. All of them reported that their disease was well controlled with medicine without any complication. No one participated in water-based exercises such as swimming during the study.

The mean wear time was 22.4 ± 2.6 hours per day. None of the participants was reported to have reached vigorous or very vigorous intensity of PA. Hence, only time spent for moderate intensity activity was included for MVPA. A total of 62 out of 63 participants (98%), 21 of 22 (96%) physicians, and 100% of nurses and supporting staff, spent over 300 min in MVPA per week meeting WHO guidelines. Among the different occupation groups, the physicians group showed significantly fewer mean counts per minute (210.4 of physicians vs. 476.0 of nurses and 441.8 of supporting staffs), MVPA per week (904.7 min of physicians vs. 1471.3 min of nurses and 1451.0 min of supporting staffs), and step counts per week (69,029 of physicians vs. 84,700 of nurses and 87,119 of supporting staffs) than the other two groups (*p* < 0.001). Nurses and supporting staff did not show a statistical difference with the above parameters. The maximal average MET per hour, time spent on the light intensity of physical activity (LPA), and calorie expended per hour was not significantly different among the occupation (Table 2 and Table 3).

Regarding the groups according to BMI, there were statistically significant differences of calories expended per hour and maximum average MET per hour among all groups (*p* < 0.001). Calorie expended per hour was 55.01, 41.44, and 27.19 and maximum average MET per hour was 3.0, 2.3, and 1.7 in over-, normal, and under-weighted groups, respectively. However, the mean counts per minute, time spent on MVPA and LPA, and step count per week were not statistically different among the groups (Table 2 and Table 3).

There was no significant difference in their mean counts per minute, time spent for MVPA and LPA, average calories expended per hour, and step counts according to sex and marital status. Men showed a significantly higher maximum average MET per hour than women (*p* < 0.05). The participants raising children burned significantly more calories per hour than those without children (1088.30 vs. 849.63 kcal/hour, *p* = 0.037) (Table 3).

**Figure 1:**
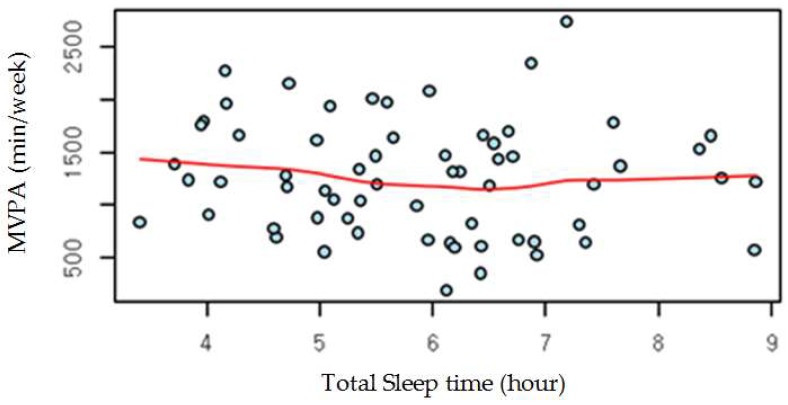
Correlation between total sleep time and moderate to vigorous intensity physical activity (MVPA).

Nineteen participants reported that they performed vigorous intensity physical exercise over 20 min per day at least once a week (0.75 ± 1.44 days). Thirty-six participants reported the completion of moderate intensity PA over 30 min per day, at least once a week (1.43 ± 1.71 days). All the participants reported having done LPA over 30 min per day, at least once a week (5.95 ± 1.57 days) on a questionnaire. All the participants showed sleep latency within the normal range (less than 30 min). Total sleep time was 5.88 ± 1.34 hours. Sixty-two of 63 participants showed normal sleep efficiency which is over 85% (91.99 ± 3.30). The total sleep time revealed no statistical difference among the occupation groups (Table 4). Furthermore, there was no correlation between total sleep time and MVPA (Figure 1).

Sixteen subjects agreed to wear the device for an additional week after receiving feedback of PA of the previous week and counseling. Their mean age was 34.2 ± 7.4 years old. Compared with the initial data, the total wear time and light intensity activity per minute, defined as total time in LPA divided by the total wear time was significantly decreased (*p* < 0.05) (Table 5). No participant did vigorous intensity of PA during the subsequent study period. The mean counts per minute, maximum average MET per hour, average calories expended per hour, moderate intensity activity per minute, defined as total time in moderate intensity of PA divided by total wear time, and step counts per minute, which is defined as the total number of steps divided by total wear time did not show significant difference before versus after counseling.

## 4. Discussion

This study provides the PA status of healthcare personnel using the accelerometer and how various factors, such as sex, occupation, marital status, having kids or not, and BMI effect the parameters of PA. Unexpectedly, the time spent in MVPA of the most of healthcare personnel met WHO guidelines [1,25]. Although 30% of participants (16 of 63) reported that they had engaged in vigorous activity for 0.75 ± 1.44 days, no one did vigorous intensity activity in data gathered by an accelerometer. This mismatch suggests that the PA measured by the questionnaire is quite subjective and may not be accurate [26]. Also, only 57% of participants (36 of 63) recalled having done MVPA for 2.17 ± 2.83 days, but nearly all of the participants revealed to fulfill MVPA recommendations.

This study shows each person’s task in the workspace would be the key factor that determines one’s amount of PA. The physician group showed significantly less time spent for MVPA, mean counts per minute, and step count per week, compared to the nurse or supporting staff groups. This might be due to desk-based tasks, aa most of them were in the non-surgery department. The surgical doctors were reluctant to participate because it was cumbersome to wear the device during surgery. Therefore, we cannot exclude the possibility of a selection bias in the physician group. Clemes et al. measured PA among office workers using an accelerometer. It showed time in MVPA was 32 ± 26 min per day [27]. The mean time in MVPA of healthcare personnel in this study is 181 ± 77 min per day, about five times more than office workers, although only 57% (36/63) recalled having engaged in MVPA during the study period. This explains the higher intensity of physical loading in hospital workers.

The high intensity of labor with a long work time is an inevitable character of tasks in hospitals. Patient handling or transferring, moving medical equipment, walking around to care for various inpatients or outpatients, and coping with the emergency are included in the tasks. The United States Department of Labor 2005 revealed a higher musculoskeletal disorder rates among healthcare practitioners [28]. Lorusso, A. reviewed musculoskeletal disorders among nursing personnel of whom 33% to 86% had low back pain [29]. These studies are in line with our results showing that healthcare personnel deal with physically high intensity tasks.

Concerning gender, men showed higher calorie expended per hour than women, whereas women had higher step counts, mean counts per minute and time in MVPA, but neither was statistically significant. The married group showed a tendency toward higher PA and calorie expended per hour, number of steps, and mean counts per minute and time in MVPA than the single group, irrespective of gender, though it was not statistically significant. Interestingly, the group who raised children showed significantly higher calorie expended per hour than the others, which may be due to additional housework.

Additionally, we aimed to figure out if the extent of PA would change after feedback and counseling. However, there was a significantly decreased wear time during the second time given that hot and humid weather might hinder one from wear the device all day long. Again, nobody did vigorous exercise. In the secondary study, all the parameters showing PA were not improved. The participants became aware of a lack of vigorous activity and recognized the need for PA; however, lifestyle modification or effort to exercise was not to be seen in a week. This result suggests that single counseling or encouraging is insufficient to lead to a prompt change in PA. There have been many studies that reveal the effective ways to increase PA. In a study, internet-based PA intervention, with self-monitoring, personalized goal setting, and interaction with other participants and study staff, was conducted on women with a family history of breast cancer. It revealed significantly increased MVPA at 3 months and 5 months [30]. Another study showed that setting a personalized target to reach an average step count per day using a pedometer was significantly effective to improve PA in patients with chronic obstructive pulmonary disease [31]. Compared with our study, their interventions were done with more specific and personalized goals. We offered to counsel and emphasized the importance of enhancing PA and subsequent health effects, but no specific goal to improve their PA was given. Also, our participants were all healthy and relatively young without underlying severe diseases, and their concern for health might not be as much as those with illnesses. Besides, the time spent working, including commute time, was almost half a day and the participants were probably too busy to make time for exercise. It seems that both internal and external factors such as motivation and work loading are obstacles to improving PA.

Many people state ‘lack of time’ as a major barrier for exercise. Over 50% of employees reported that they have little time to exercise because of a busy work schedule [32]. Also, a busy home or family schedule was a barrier for nearly 50% of participants [32]. Besides, over 75% of workers are employed in sedentary jobs [10]. Therefore, increasing PA in the workplace can be one solution to enhance PA. Due to the nature of hospital tasks, most of the healthcare personnel meet the MVPA criteria of recommendation, but enhancing PA among office workers is not easy. Nevertheless, some studies have been conducted to increase PA in the workplace. John et al. performed an empirical study of treadmill workstations—sedentary office-workers were made to work while walking. It revealed that standing, stepping time and total steps per day were significantly increased. Also, lipid profile and the circumference of the hip and waist were improved [33]. Another study included the intervention strategies introduced was a workplace walking program which encouraged walking instead of sitting during work tasks [34]. Other study assigned their participants a mandatory activity of middle-to-high intensity for 2.5 h per week during their work hours [35]. However, whether the above-mentioned strategies are effective in promoting PA is questionable since their evidence proved to be insufficient or less significant [36]. More effective and attractive strategies to get the participants involved in PA at their workplaces are promised in the future.

This study has several limitations. First, only a small number of the workers participated. The number of participants was smaller than expected which may be due to less interest in PA and the cumbersome of wearing the device in a hospital working environment. Especially, the number of the under-weight group was four, and this is too small a sample size to interpret as conclusive. The study was exploratory research to observe the PA status of workers in a hospital, hence, power calculation was not done initially. However, there have been a few studies on the PA of healthcare personnel, therefore this study is meaningful that our results show PA status of healthcare personnel with an objective manner. Further research with a more significant number of participants is needed for more in-depth analysis. Second, the working hours of the subjects were not consistent, which is a characteristic of healthcare personnel. Their working time was divided into day and night time, including the three eight-hour shift schedules of nurses. Additionally, because of the small number of participants, data during work time and off-duty time were not considered. Despite these limitations, we were able to study PA based on each’s characteristics within one common workplace.

## 5. Conclusions

Most of the participants fulfilled the recommended level and amount of PA despite the lack of vigorous intensity of PA. This implicates that the physical intensity of tasks of the hospital is high and health care workers are not much involved in leisure activity for individual exercise. Among subgroups, the physicians revealed to have significantly less PA compared to nurses or supporting staff. Gender and marital status did not influence the PA of workers in the hospital. However, raising a child may increase the average calorie expenditure. Also, individual counseling, including education on the positive impact and necessity of PA was not effective towards increasing PA. This shows that a single intervention without an individualized goal and checkup might be insufficient to lead to a successful outcome. To improve health, we need to constantly pay attention to increase PA in daily life, including in the workplace. Future research should focus on practical ways to enhance PA, including strategies which can be applied in the workplace.

## Figures and Tables

**Table 1:** Demographic characteristics of the participants.

Variable	No. of the Participants
Sex	
Male	31
Female	32
Occupation	
Physician	22
Nurse	18
Supporting staffs	23
Marital status	
Married	34
Unmarried	29
Have kids	
With kids	30
With no kids	33
BMI (kg/m^2^)	
Under-weighted (BMI < 18.5)	4
Normal weighted (18.5 ≤ BMI < 23)	38
Over-weighted (BMI ≥ 23)	21
Smoking	
Non-smoker	54
Smoker	9
Medical history	
None	56
Hypertension	5
Diabetes	1
Hyperthyroidism	1

**Table 2:** Physical activity of the participants.

Variable	Intensity of activity							
	Light Intensity Activity per Week (min)		MVPA per Week (min)		Mean Counts per Minute		Maximum Average MET per Hour	
	Mean (SEM)	*p-*Value	Mean (SEM)	*p-*Value	Mean (SEM)	*p-*Value	Mean (SEM)	*p-*Value
Sex								
Male	3329.5 (151.3)	0.451	1257.6 (99.7)	0.904	360.4 (45.0)	0.74	3.0 (1.0)	0.00
Female	3481.2 (131.6)		1274.2 (95.3)		380.9 (41.7)		2.0 (0.1)	
Occupation								
Physician	3519.4 (190.6)	0.435	904.7 (82.3)	0.00 ^†^	210.4 (31.8)	0.00 ^†^	2.4 (0.2)	0.159
Nurse	3401.4 (119.0)		1471.3 (128.0)		476.0 (59.9)		2.4 (0.2)	
Supporting staffs	3302.5 (184.3)		1451.0 (106.1)		441.8 (48.0)		2.7 (0.1)	
Marital status								
Married	3503.3 (143.7)	0.297	1298.2 (88.0)	0.614	374.1 (38.7)	0.908	2.6 (0.1)	0.075
Unmarried	3293.0 (135.8)		1228.3 (108.2)		366.9 (48.8)		2.3 (0.1)	
Have kids								
With kids	3440.2 (142.9)	0.75	1324.6 (98.7)	0.418	387.9 (43.2)	0.596	2.6 (0.1)	0.186
Without kids	3375.9 (141.0)		1212.8 (95.2)		355.3 (43.2)		2.4 (0.1)	
BMI (kg/m^2^)								
Under-weighted (BMI < 18.5)	3613.8 (266.9)	0.672	818.8 (156.3)	0.205	187.0 (67.6)	0.219	1.7 (0.1)	0.00 ^‡^
Normal weighted (18.5 ≤ BMI < 23)	3437.7 (121.0)		1296.3 (87.6)		386.0 (38.3)		2.3 (0.1)	
Over-weighted (BMI ≥ 23)	3310.6 (201.0)		1296.5 (122.6)		378.4 (56.8)		3.0 (0.1)	

^†^ Significantly different between physicians and nurses or supporting staff, ^‡^ significantly different among all groups (*p* < 0.05).

**Table 3:** Physical activity of the participants.

Variable	Average Kcal per Hours (kcal)		Steps Counts per Week		Sedentary Activities (%)	Light + Moderate Intensity Activities (%)		Vigorous and Very Vigorous Intensity Activity (%)
	Mean	*p-*Value	Mean	*p-*Value	Mean	Mean	*p-*Value	
Sex								
Male	1050.67	0.137	78,959	0.701	51.36	48.64	0.455	0
Female	878.63		81,227		49.52	50.48		0
Occupation								
Physician	951.06	0.123	69,029	0.01 ^†^	50.77	49.23	0.616	0
Nurse	900.66		84,700		48.55	51.45		0
Supporting staffs	1023.90		87,119		51.56	48.44		0
Marital status								
Married	993.37	0.6	81,252	0.695	48.99	51.01	0.234	0
Unmarried	932.22		78,934		51.91	48.09		0
Have kids								
With kids	1088.30	0.037	83,698	0.245	49.09	50.91	0.3	0
Without kids	849.63		76,850		51.64	48.36		0
BMI (kg/m^2^)								
Under-weighted (BMI < 18.5)	27.19	< 0.001 ^‡^	66,340	0.288	53.39	46.61	0.48	0
Normal weighted (18.5 ≤ BMI < 23)	41.44		81,051		50.36	49.64		0
Over-weighted (BMI ≥ 23)	55.01		81,032		49.93	50.03		0

^†^ Significantly different between physicians and nurses or supporting staff, ^‡^ significantly different among all groups, *p* < 0.05.

**Table 4:** Exercise reported with questionnaire and sleep acquired by accelerometer.

Variable	Mean ± SD	*p*-Value
Exercise record by questionnaire (No. (d))		
Light intensity	63 (5.95 ± 1.57)	
Moderate intensity	36 (1.43 ± 1.71)	
Vigorous intensity	19 (0.75 ± 1.44)	
Moderate to vigorous intensity	36 (2.17 ± 2.83)	
Total Sleep		
Sleep time (h)	5.88 ± 1.34	
Efficiency (%)	91.99 ± 3.30	
Sleep time (h)		
Physician	5.55 ± 1.11	0.27
Nurse	6.24 ± 1.36	
Supporting staffs	5.92 ± 1.49	

**Table 5:** Comparison of physical activity before and after counseling.

Test	Mean (SEM)							
	Total Wear Time per Day (hr)	Mean Counts per Minute	Maximum Average MET per Hour	Light Intensity Activity per Minute *	Moderate Intensity Activity per Minute *	Vigorous and Very Vigorous Intensity Activity (%)	Average Calorie Expended per Hours (kcal)	Average Steps Count per Minute ^†^
Initial	21.9 (0.8)	395.7 (72.4)	2.6 (0.1)	0.34 (0.03)	0.14 (0.02)	0	46.53	8.3
After counseling	16.2 (1.1)	413.5 (100.9)	2.6 (0.2)	0.25 (0.02)	0.13 (0.02)	0	38.25	7.6
*p*-Value	0.003	0.975	0.594	0.03	0.79	-	0.23	0.334

* Light/moderate intensity activity per minute is defined as the total time in light/moderate intensity of physical activity divided by total wear time. ^†^ Average step count per minute is defined as the total number of steps divided by total wear time.
